# Characterization of the Interaction between Gallic Acid and Lysozyme by Molecular Dynamics Simulation and Optical Spectroscopy

**DOI:** 10.3390/ijms160714786

**Published:** 2015-07-01

**Authors:** Minzhong Zhan, Ming Guo, Yanke Jiang, Xiaomeng Wang

**Affiliations:** 1 School of Science, Zhejiang Agricultural & Forestry University, Lin’an 311300, China; E-Mails: manuscript.usezl@gmail.com (M.Z.); wangxiaomeng0426@163.com (X.W.); 2 Research Center of Medical Chemistry & Chemical Biology, Chongqing Technology and Business University, Chongqing 400067, China; E-Mail: ctbujyk@swu.edu.cn

**Keywords:** gallic acid, lysozyme, molecular dynamics simulation, spectroscopic techniques, MM-PBSA method

## Abstract

The binding interaction between gallic acid (GA) and lysozyme (LYS) was investigated and compared by molecular dynamics (MD) simulation and spectral techniques. The results from spectroscopy indicate that GA binds to LYS to generate a static complex. The binding constants and thermodynamic parameters were calculated. MD simulation revealed that the main driving forces for GA binding to LYS are hydrogen bonding and hydrophobic interactions. The root-mean-square deviation verified that GA and LYS bind to form a stable complex, while the root-mean-square fluctuation results showed that the stability of the GA-LYS complex at 298 K was higher than that at 310 K. The calculated free binding energies from the molecular mechanics/Poisson-Boltzmann surface area method showed that van der Waals forces and electrostatic interactions are the predominant intermolecular forces. The MD simulation was consistent with the spectral experiments. This study provides a reference for future study of the pharmacological mechanism of GA.

## 1. Introduction

At present, targeted drug delivery that directly leads to drug delivery to target organs, tissue, and cells is a modern pharmaceutical research hotspot. Ligand-receptor-mediated active targeting drug delivery systems have received much attention in recent years [1,2]. However, studies about the interactions between drug and target molecules have mostly concentrated on serum protein, and other molecular targets are rarely reported [3,4,5]. Investigations of the interactions between different target molecules and drugs are of great significance for comprehensive understanding of drug transport and metabolism processes. Enzymes are a type of bioactive substance with efficient catalytic ability. Almost all complex and regular activities of organisms cannot occur without enzymes. Enzymes are important carriers of drug efficacy exertion and target molecules *in vivo*. The properties of enzymes are a hot research field in biochemistry [6,7,8].

**Figure 1:**
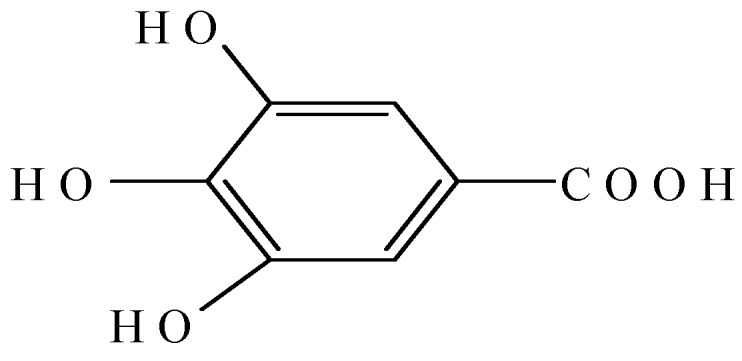
Molecular structure of gallic acid.

Lysozyme (LYS) is a single-chain protein composed of 129 amino acid residues that widely exists in organisms. LYS can combine with many endogenous and exogenous substances to exert its antibacterial, anti-inflammatory, and antitumor properties [9]. LYS has been widely used as a model protein in studies of enzyme kinetics and enzyme activity [10,11]. Spectroscopic methods are commonly used to investigate the interaction between LYS and drugs [12,13,14]. Gallic acid (GA), also known as 3,4,5-trihydroxybenzoic acid, is a polyphenolic compound that widely exists in nature, such as in rhubarb, polygonum multiflorum, peony, other herbs, grapes, strawberries, pineapples, and guava fruit tissues [15]. The molecular structure of GA is shown in Figure 1. GA is a natural anticancer drug with many biological activities, such as anti-inflammatory, antitumor, antimutation, and antioxidant [15,16]. It is also an important organic fine chemical product, which is widely used in agriculture, daily chemical products, food, and paints [16,17].

Molecular mechanics and molecular dynamics (MD) simulations are now being used to understand the complex process of guest-host interactions [18,19,20,21,22]. Recently, the molecular mechanics/Poisson-Boltzmann surface area (MM-PBSA) approaches have emerged as effective computational approaches to calculate protein-ligand binding free energies based on MD of the given protein–ligand complex in explicit solvent [23,24,25,26,27].

To the best of our knowledge, the binding mechanism between GA and LYS has not been reported. In this study, the binding between GA and LYS was investigated to understand the specific interaction mechanism. This research is important to understand the distribution and metabolism of GA *in vivo*, and to clarify the relationship between the structure and function of LYS. In addition, the interaction model is an important reference to further expand the drug-target interaction field. Here, fluorescence and UV-vis spectroscopy were used to obtain information about the binding mechanism of GA to LYS. The MD technique was used to explain the interaction between GA and LYS at a molecular level based on spectroscopic data. The information obtained in this study could help to shed light on the interaction mechanism between GA and LYS. It could also guide the design of new drugs for the treatment of a variety of diseases associated with GA [28,29].

## 2. Results and Discussion

### 2.1. Fluorescence and UV–vis Spectroscopy

**Figure 2:**
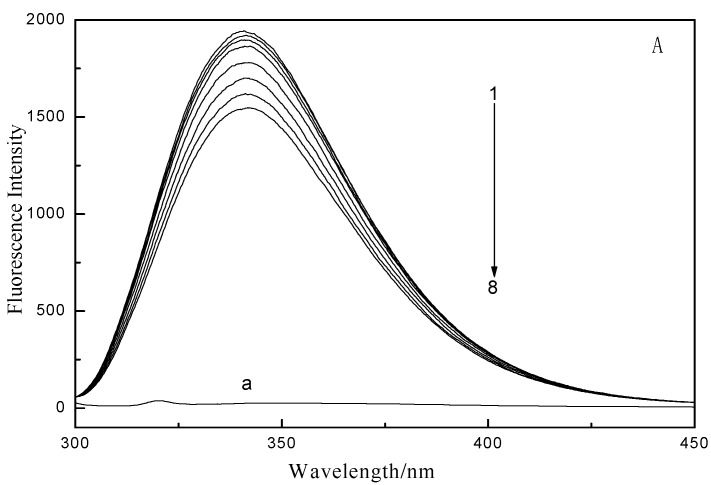

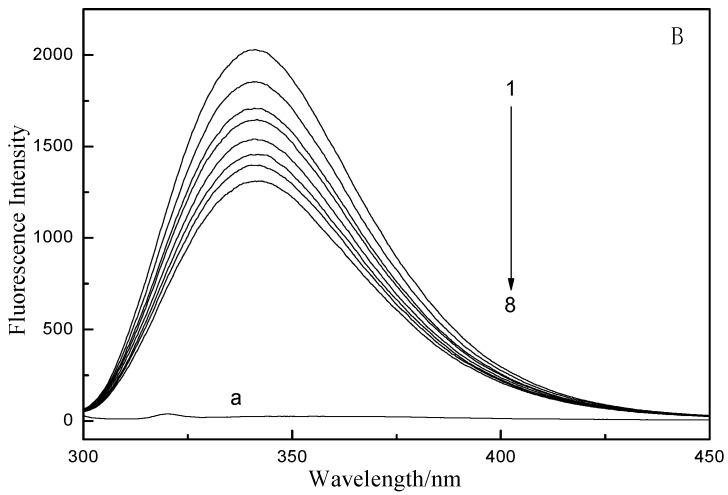
Fluorescence quenching spectra of gallic acid effect on lysozyme (**A**) 298 K; (**B**) 310 K. Curves (1–8): A series 1.0 mL LYS solution (1.0 × 10^−5^ mol/L) were titrated by adding GA stock solutions with a concentrations from top to bottom were 0.0, 0.4, 0.8, 1.0, 1.6, 2.4, 3.2 and 4 × 10^−5^ mol/L. a: The concentration of GA was 1.0 × 10^−5^ mol/L. The fluorescence spectra of GA-LYS system were recorded using a F-7000 spectrofluorophotometer (Hitachi Co., Ltd., Tokyo, Japan) at emission wavelength range of 250–500 nm with λ_ex_ = 282 nm and the slit widths were 2.5 nm.

The interaction between GA and LYS was investigated by fluorescence and UV-vis spectroscopy. The fluorescence of LYS originates from tryptophan (Trp), tyrosine (Tyr), and phenylalanine (Phe) residues, and the main contribution to the intrinsic fluorescence of LYS is Trp residues. The fluorescence spectra of LYS (Figure 2) showed that the LYS fluorescence intensity slightly decreased after addition of GA. The maximum emission of LYS was 341 nm upon excitation at 282 nm. Moreover, with increasing GA concentration, the fluorescence intensity of LYS progressively decreased but the emission maximum wavelength did not change. The fluorescence result indicates that GA has no fluorescence intensity, and also that the polarity microenvironment of LYS changed. Addition of GA to the solution of LYS resulted in quenching of its fluorescence emission because of change of the microenvironment around the amino acid residues. The fluorescence intensity of LYS did not increase with the presence of GA, indicating that the energy change between the two is a Förster’s nonradiative energy transfer process.

**Figure 3:**
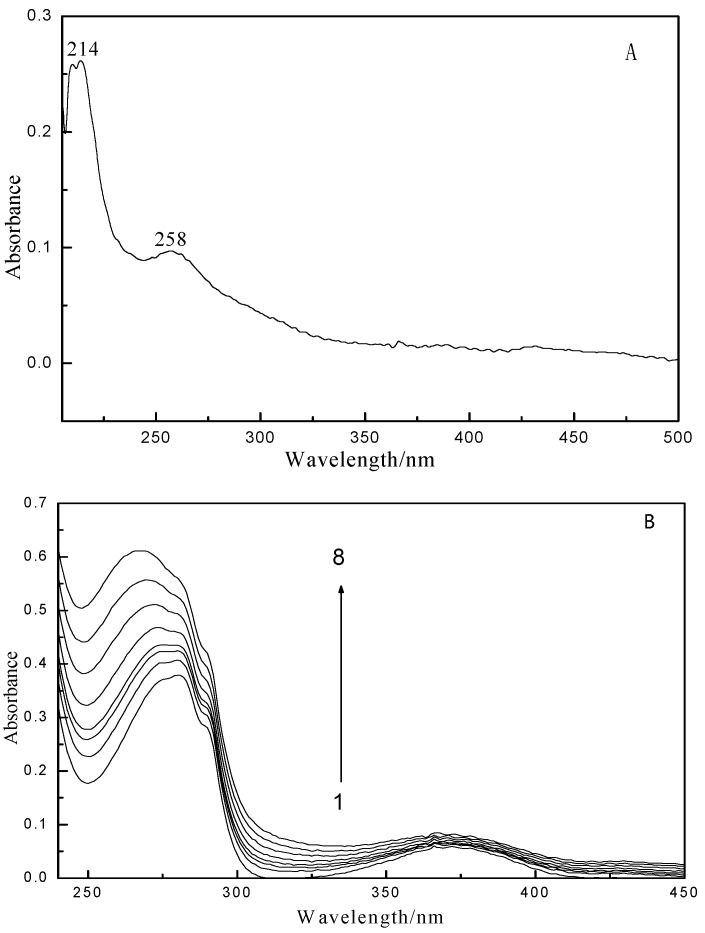
UV absorption spectrum of GA (**A**) and GA-LYS (**B**). Curves (1–8): A series 1.0 mL LYS solution (1.0 × 10^−5^ mol/L) were titrated by adding GA stock solutions with a concentrations from top to bottom were 0.0, 0.4, 0.8, 1.0, 1.6, 2.4, 3.2 and 4 × 10^−5^ mol/L. The UV absorption spectra of GA and GA-LYS system were recorded using a UV-2450 UV-vis spectrometer (Shimadzu Co., Kyoto, Japan).

UV-vis absorption spectroscopy is a simple but efficient technique to investigate structural changes and complex formation. GA can absorb at either an excitation wavelength of 282 nm or a LYS emission wavelength of 341 nm, as shown in the GA ultraviolet absorption spectrum (Figure 3A). Figure 3B shows the UV absorption spectra of LYS in the absence and presence of GA. To correct for the inner filter effects caused by GA and LYS, Equation (1) [30,31,32] was used to correct the fluorescence intensity.

(1) $$F_{\text{corr}}=F_{\text{abs}}\times 10^{\frac{A(\lambda_{\text{exc}})}{2}}\times 10^{\frac{A(\lambda_{\text{em}})}{2}}$$

where *F*_corr_ is the correct fluorescence intensity, *F*_abs_ is the measured fluorescence intensity, and *A*(λ_ex_) and *A*(λ_em_) are the absorbance value at excitation and emission, respectively. The fluorescence intensity values used in this paper are the fluorescence values after correction.

### 2.2. Fluorescence Quenching Mechanism

The fluorescence quenching mechanism is usually divided into static quenching and dynamic quenching [33]. The dynamic quenching mechanism can be described by the Stern-Volmer equation [34]. The present work aimed to investigate whether GA interacts with LYS, and to identify the quenching mechanism. To obtain the quenching mechanism, the procedure was assumed to be a dynamic quenching and fluorescence quenching, and data were disposed at two different temperatures. The Stern-Volmer equation [35,36,37,38] is

(2) $$F_{0}/F=1+K_{q}\tau_{0}[Q]-1+K_{sv}[Q]$$

where *K_q_*, *K_sv_*, τ_0_, and [*Q*] are the quenching rate constant of the biomolecule, the dynamic quenching constant, the average lifetime of the molecule without quencher, and the concentration of quencher, respectively. The dynamic quenching parameters of GA and LYS (*K_sv_*) can be obtained from the experimental data using the Stern-Volmer equation. The fluorescence lifetime of the biopolymer is about 10^−8^ s [30,39,40], so the quenching constant (*K_q_*, L/(mol·s)) can be obtained from *K_q_* = *K_sv_*/τ_0_.

**Figure 4:**
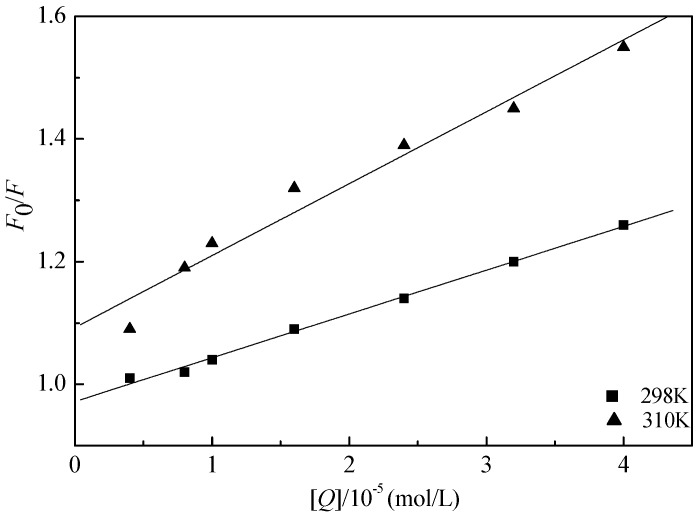
Stern-Volmer plot for the fluorescence quenching of LYS by GA (298 and 310 K).

Table 1 and Figure 4 show the results and fitting curve, respectively. The plots show good linear relationships, suggesting that a single type of quenching phenomenon (either static or dynamic quenching) occurs in the formation of GA-LYS complexes. Because the maximum scatter collision quenching constant *K_q_* of various quenchers with the biopolymer is 2.0 × 10^10^ L/(mol·s) [30,41] and the rate constant of the protein quenching procedure initiated by GA is much greater than the *K_q_* value of the scatter procedure, quenching is not initiated by dynamic quenching but by compound formation. In addition, the ultraviolet absorbance intensity changed markedly with increasing drug concentration (Figure 3), which indicates a static quenching mechanism [42,43].

**Table 1:** The quenching constants and binding parameters of LYS to GA solution system.

*T* (K)	*K*_sv_ (L/mol)	*K*_q_ (L/(mol·s))	*R*	*SD*	*p*
298 K	7.15 × 10^3^	7.15 × 10^10^	0.9980	0.0066	<0.0001
310 K	1.17 × 10^4^	1.17 × 10^12^	0.9829	0.0321	<0.0001

### 2.3. Binding Mechanism and Thermodynamic Parameters

For the static quenching interaction, if it is assumed that there are similar and independent binding sites in the biomolecule, the binding constant (*K*) and the number of binding sites (*n*) can be determined according to the following equation [44]:

(3) $$\log[(F_{0}-F)/F]=\log K+n\log[Q]$$

where *F*_0_, *F*, and [*Q*] are the same as those in Equation (2), *K* is the binding constant of GA with LYS, and *n* is the number of binding sites per LYS molecule, which can be determined by the slope and intercept of the double logarithm regression curve of log[(*F*_0_ − *F*)/*F*] *versus* log[*Q*] based on Equation (3). Table 2 shows the values of *K* and *n* for LYS at 298 and 310 K determined in this way. The *n* values of GA-LYS complexes are approximately equal to 1, indicating that there is one binding site in LYS for GA. The binding constant *K* (Table 2) decreased with increasing temperature, showing that the binding between GA and LYS gets weaker as the temperature gets warmer.

Generally, small molecules bind to biological macromolecules by a combination of hydrogen bonding, van der Waals, electrostatic, and hydrophobic interactions. The signs and magnitudes of the thermodynamic parameters (e.g., enthalpy change (Δ*H*°) and entropy change (Δ*S*°)) associated with the drug-protein interaction process can be used to determine the binding force(s) [45,46]. For this reason, the thermodynamic parameters were investigated, and they are calculated by the Van’t Hoff equation:

(4) $$\ln K=\frac{-\Delta H^{{}^{\circ}}}{RT}+\frac{\Delta S^{{}^{\circ}}}{R}$$

(5) $$\Delta G^{{}^{\circ}}=\Delta H^{{}^{\circ}}-T\Delta S^{{}^{\circ}}$$

where *R* is the universal gas constant, *K* is the binding constant at temperature *T*, and *T* is the temperature in kelvin (291 and 310 K). Briefly, the interaction can be summarized based on of the thermodynamics data as follows [36,47]:

(i) Δ*H*° > 0 and Δ*S*° > 0 corresponds to hydrophobic forces.

(ii) Δ*H*° < 0 and Δ*S*° < 0 corresponds to van der Waals interactions and hydrogen bond formation.

(iii) Δ*S*° > 0 corresponds to electrostatic interactions or hydrophobic forces.

**Table 2:** Binding parameters and thermodynamic parameters of LYS to GA interaction system.

*T* (K)	*K* (L/mol)	*n*	Δ*G* (*k*J/mol)	Δ*S* (*k*J/(mol·K))	Δ*H* (*k*J/mol)
298	3.93 × 10^5^	1.3985	−31.92	−1.20	−388.81
310	9.04 × 10^2^	0.7274	−17.54	−1.20	−388.81

The calculated thermodynamic parameters are shown in Table 2. Because both Δ*H*° and Δ*S*° are less than zero, it is concluded that van der Waals interactions and hydrogen bond formation are the predominant driving forces for GA-LYS binding.

### 2.4. Energy Transfer from GA to LYS

**Figure 5:**
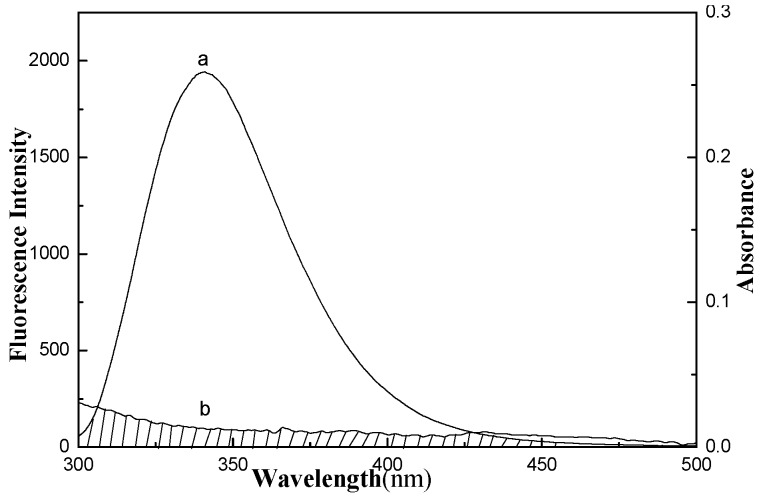
Overlapping between the fluorescence emission spectra of LYS (a) and UV absorption spectra of GA (b).

The efficiency of energy transfer (*E*) is calculated according to Förster’s nonradioactive energy transfer theory [48]. It can be calculated from donor emission and acceptor absorption spectra using:

(6) $$E=1-\frac{F}{F_{0}}=\frac{R_{0}^{6}}{\left(R_{0}^{6}+r^{6}\right)}$$

(7) $${R_{0}}^{6}=8.8\times 10^{-25}K^{2}N^{-4}\Phi J$$

where *r* is the distance from the ligand to the protein, *R*_0_ is the Förster critical distance at which 50% of the excitation energy is transferred to the acceptor, *K* is the spatial orientation factor of the dipole (*K*^2^ = 2/3) [49], *N* is the refractive index of the medium (*N* = 1.336), *Φ* is the fluorescence quantum yield of the donor (*Φ* = 0.118), and *J* is the overlap integral of the fluorescence emission spectrum of the donor and that of the absorption spectrum of the acceptor. Therefore,

(8) $$J=\int_{0}^{\infty }F(\text{λ})\text{ε(λ}){\text{λ}}^{4}d\text{λ}/\int_{0}^{\infty }F(\text{λ})d\text{λ}$$

where *F*(λ) is the fluorescence intensity of the fluorescent donor at wavelength λ, ε(λ) is the molar absorptivity of the acceptor at wavelength λ. According to Figure 5 and formula 8, the following data were obtained for GA-LYS system: *E* = 0.203, *J* = 1.15 × 10^−15^ cm^3^·L/mol, *R*_0_ = 1.69 nm, and *r* = 2.12 nm. Obviously, the donor–acceptor distance (*r*) is less than 6 nm~3R_0_ nm [34], suggesting that there is a high probability of nonradiative energy transfer from LYS to GA. The distance values here is the distance between the fluorophore (tryptophan, tyrosine and phenylalanine) and the ligand, it is a theoretical value.

### 2.5. Conformation Investigation

**Figure 6:**
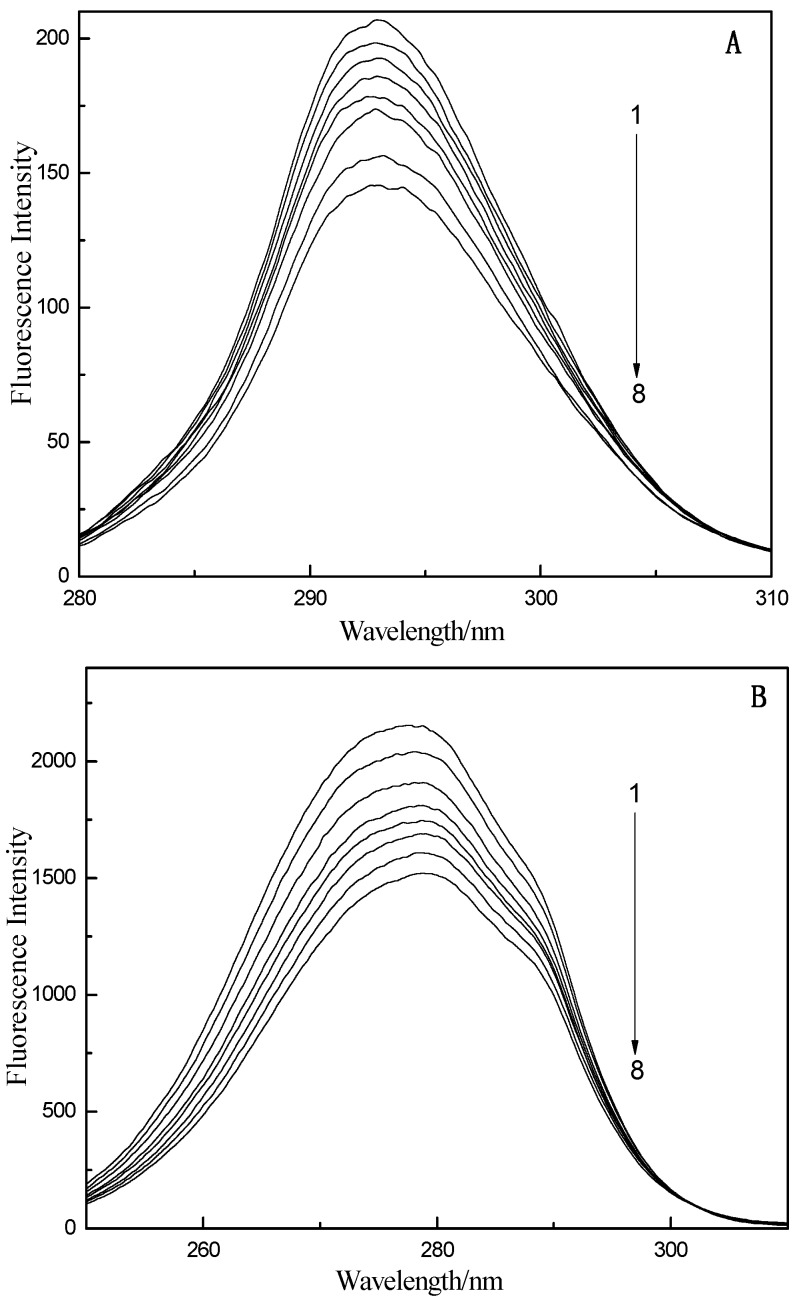
Effect of GA on the synchronous fluorescence spectra of LYS ((**A**) Δλ = 15 nm; (**B**) Δλ = 60 nm). *c*_LYS_ = 1.0 × 10^−5^ mol/L, *c*_GA_(1 to 8) = 0.0, 0.4, 0.8, 1.6, 2.8, 3.6, 4.8, 5.6 (×10^−5^ mol/L).

Synchronous fluorescence spectroscopy and the fluorescence phase diagram method were used to further investigate the structural change of LYS with increasing GA concentration. The conformational changes of LYS were evaluated by measuring the synchronous fluorescence intensity (at different scanning intervals (Δλ = λ_em_ − λ_ex_)) of the protein amino acid residues before and after addition of GA. When Δλ = 15 nm, the spectrum characteristic of the protein Tyr residues is observed, and when Δλ = 60 nm, the spectrum characteristic of protein Trp residues is observed [50]. Figure 6A,B show the fluorescence intensity of LYS for Δλ = 15 and 60 nm, respectively, which both decrease with increasing GA concentration. No obvious shift in the Δλ = 15 nm emission maximum upon quenching was observed (Figure 6A), but the emission wavelength of the Trp residues red-shifted (1.6 nm) (Figure 6B) with increasing GA concentration. These suggest that the interaction of GA with LYS does not affect the conformation of the Tyr microregion. But the Trp fluorescence may indicate that the conformation of LYS changed, leading to the polarity around Trp residues increasing and the hydrophobicity decreasing. This shows that the peptide chain around the Trp residues is exposed to a more hydrophilic microenvironment, resulting in the LYS conformational change.

To further characterize the change in the dynamic morphology of LYS when GA binds, the fluorescence phase diagram method [51] was used. The fluorescence phase diagram method describes the structure change of a protein when the fluorescence intensity *I*(λ_1_) and *I*(λ_2_) of the protein change under different experimental conditions for the emission wavelengths λ_1_ and λ_2_. The fluorescence intensity can be determined as follows [52]:

(9) $$\begin{array}{l} I({\text{λ}}_{1})=a+bI({\text{λ}}_{2})\\ a=I_{1}({\text{λ}}_{1})I_{1}({\text{λ}}_{2})(I_{2}({\text{λ}}_{1})-I_{1}({\text{λ}}_{1}))/I_{2}({\text{λ}}_{2})-I_{1}({\text{λ}}_{2})\\ b=(I_{2}({\text{λ}}_{1})-I_{1}({\text{λ}}_{1}))/(I_{2}({\text{λ}}_{2})-I_{1}({\text{λ}}_{2})) \end{array}$$

where *I*(λ_1_) is the fluorescence intensities when fluorescence emission wavelengths is λ_1_ and *I*(λ_2_) is the fluorescence intensities when fluorescence emission wavelengths is λ_2_. *I*_1_(λ_1_) and *I*_2_(λ_1_) are the fluorescence intensities measured corresponding to the initial state and the terminal state of LYS on wavelengths λ_1_, *I*_1_(λ_2_) and *I*_2_(λ_2_) are the fluorescence intensities measured corresponding to the initial state and the terminal state of LYS on wavelengths λ_2_, *a* and *b* are the vertical intercept and slope of the fitting curve *I*(λ_1_) *vs.* *I*(λ_2_) respectively.

**Figure 7:**
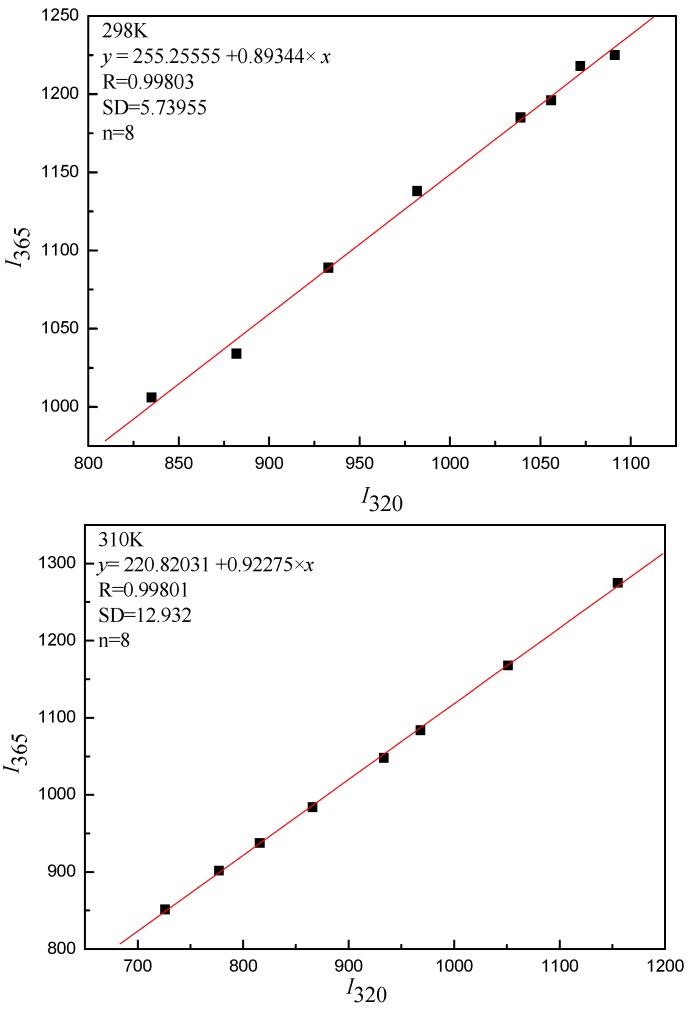
The fluorescence phase diagrams of GA-LYS. *c*_LYS_ = 1.0 × 10^−5^ mol/L, *c*_GA_(1 to 8) = 0.0, 0.4, 0.8, 1.6, 2.8, 3.6, 4.8, 5.6 (×10^−5^ mol/L).

In this paper, we compared the fluorescence phase diagram and the related coefficient of GA-LYS for multiple wavelengths, and the results indicated that the phase diagram with *I*_320_–*I*_365_ best reflects the conformation transformation process. Figure 7 shows the corresponding fluorescence phase diagram based on the experimental data. The GA and LYS fluorescent phase diagram is linear, and thus the conformation state change of LYS followed a two-state model, not a pattern change process, when GA noncovalently binds to LYS.

### 2.6. Molecular Dynamics Simulation Analysis

Molecular docking and molecular dynamics simulations were carried out to understand the specific binding position and interaction between GA and LYS. The 3D structure of LYS in complex with its 1,2-ethanediol ligand (PDB code: 1GWD) was downloaded from the PDB databse. All the Cl, I, Na and CO atoms were removed by an Autodock program. After correcting atom types and adding all the hydrogen atoms, molecular building was done for GA ligand with molecular sketch program based on the structure of 1,2-ethanediol ligand. Geometry optimization was carried out using MAXIMIN molecular mechanics and Gaussian98, and the convergence criterion set at 0.05 kcal·Å^−1^·mol^−1^ for ligands [53]. The molecular docking between GA and LYS was carried out with AutoDock4.02 running on a Silicon Graphics Ocatane 2 workstation under the Linux system [54]. The docked GA-LYS complexes were used as the initial structure for the following molecular dynamics simulation using SANDER module in AMBER.

**Figure 8:**
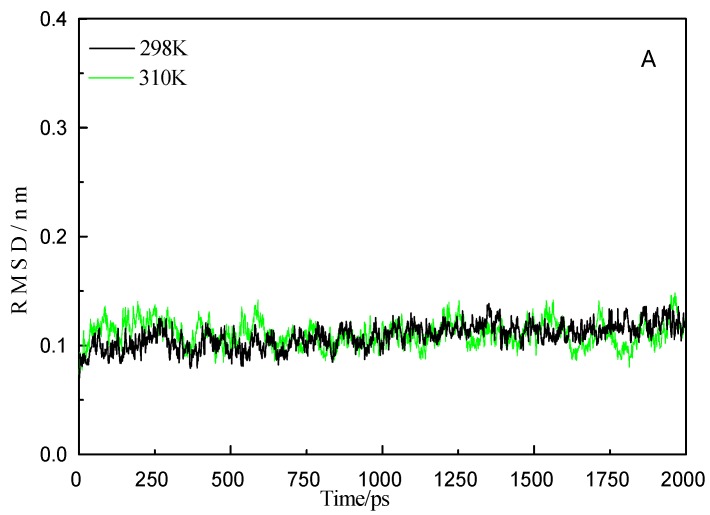

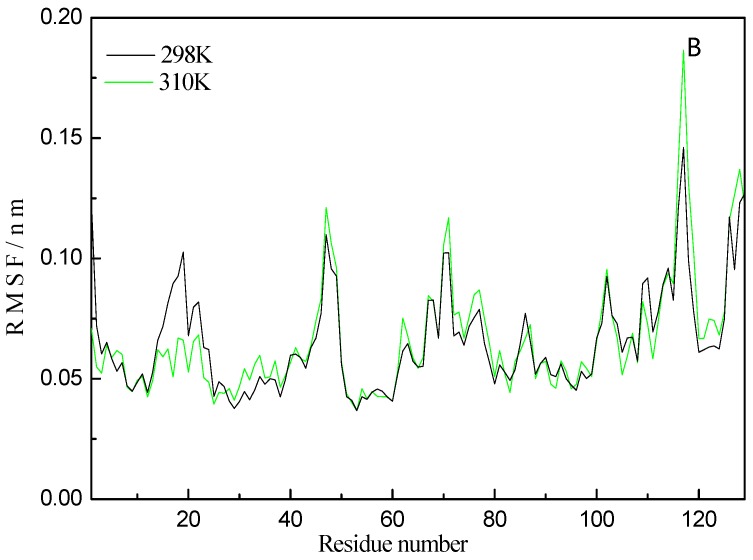
RMSD (**A**) change of receptor LYS backbone at in 2000 ps MD simulation and RMSF (**B**) fluctuation with the residues number for receptor LYS at 298 and 310 K.

The root-mean-square deviation (RMSD) values of the backbone atoms during the MD simulation were obtained. This is a way to determine whether the system is stable [53]. To observe the changes of the LYS structure, 2 ns molecular dynamics simulations were carried out on the GA-LYS system at 298 and 310 K. The curves in Figure 8A show the RMSD of C _α_ atoms as a function of time. The RMSD values of the backbone atoms of the complex rapidly increase in the first 280 ps because of protein structure optimization, and then the values are essentially stable. The change of the RMSD values at 310 K is larger than at 298 K (Figure 8A), suggesting that temperature has an influence on the interaction between GA and LYS. That is, the higher the temperature, the greater the fluctuation of the backbone atoms, which is in agreement with the spectroscopy results. The RMSD values of the two systems indicate that the conformation achieved equilibrium after 700 ps, which indicates that GA can bind to LYS to form stable compounds at both 298 and 310 K, and the complex system is stable and reasonable.

The structural changes in the MD simulation can be described by the isotropic temperature factors (B), which provide a way to determine the root-mean-square fluctuation (RMSF) of separate residues [55,56]. There are 129 residues in LYS. Figure 8B shows the RMSF of separate residues of the model at 298 and 310 K with respect to the starting structures in the 2 ns MD run. The C_α_ backbone of LYS changes differently at 298 and 310 K. The overall ratio of RMSF calculated for the residues excluding the α helix region around residues 14–27 is higher at 310 K than at 298 K. The unusual RMSF fluctuation around residues 14–27 residues indicates that the local structures may be adjusted by individual residues in and out of the α helix and β folded regions [55,57]. At both temperatures, the RMSF values are highest around residues 45–49, 67–76, and 114–118, indicating that these sections are more flexible. The active pocket composed of residues 3–12, 25–43, and 51–65 have much lower flexibility and higher stability than the other residues. The difference between two temperatures are not very big, but it is the same module under 298/310 K MD simulation; perhaps the MD temperature conditions we chose according to our experiment cannot cause significant RMSF change.

**Figure 9:**
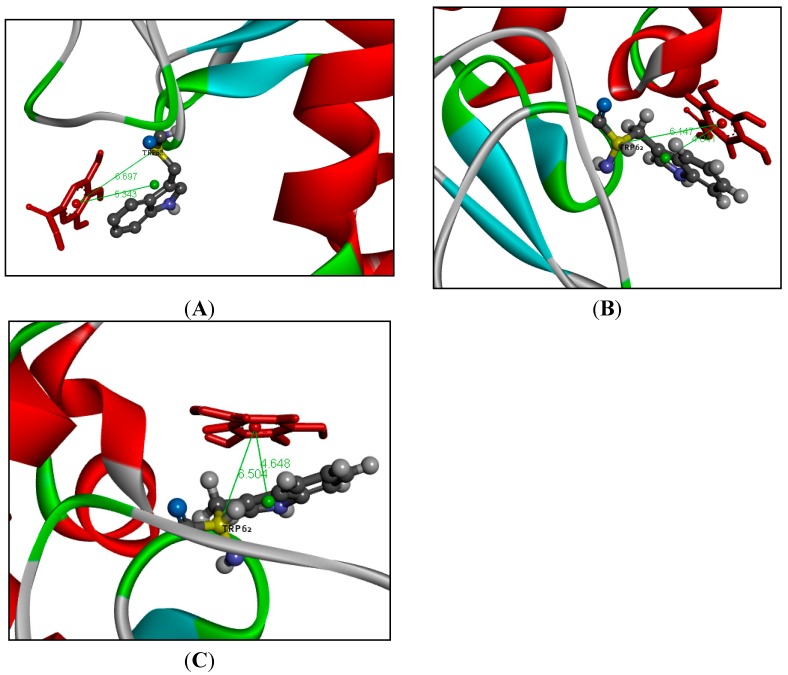
Distance map of GA to Trp62 of LYS, only picked Trp62 as a example. (**A**) The interaction mode between GA and LYS before MD simulation; (**B**) The interaction mode between GA and LYS after 298 K MD simulation; (**C**) The interaction mode between GA and LYS after 310 K MD simulations. (The ligand structure is represented using red stick model, and the red ball is the centroid of GA. The distances are remarked using green lines. The residue Trp62 is represented using a ball and stick model, the green ball represents the centroid of Trp and the yellow ball represents the C_α_ of Trp).

**Figure 10:**
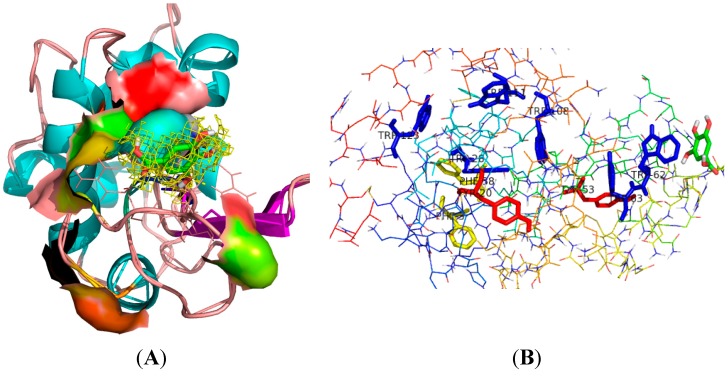
Interaction of GA with LYS. (**A**) Electron density and hydrophobic surface map between GA- LYS, only residues around 20.0 Å of the ligand are displayed; (**B**) Interaction mode between GA and LYS. The ligand structures are represented using a stick model, the blue sticks represented Trp residues, the red sticks represented Tyr residues, the yellow sticks represented Phe residues.

**Figure 11:**
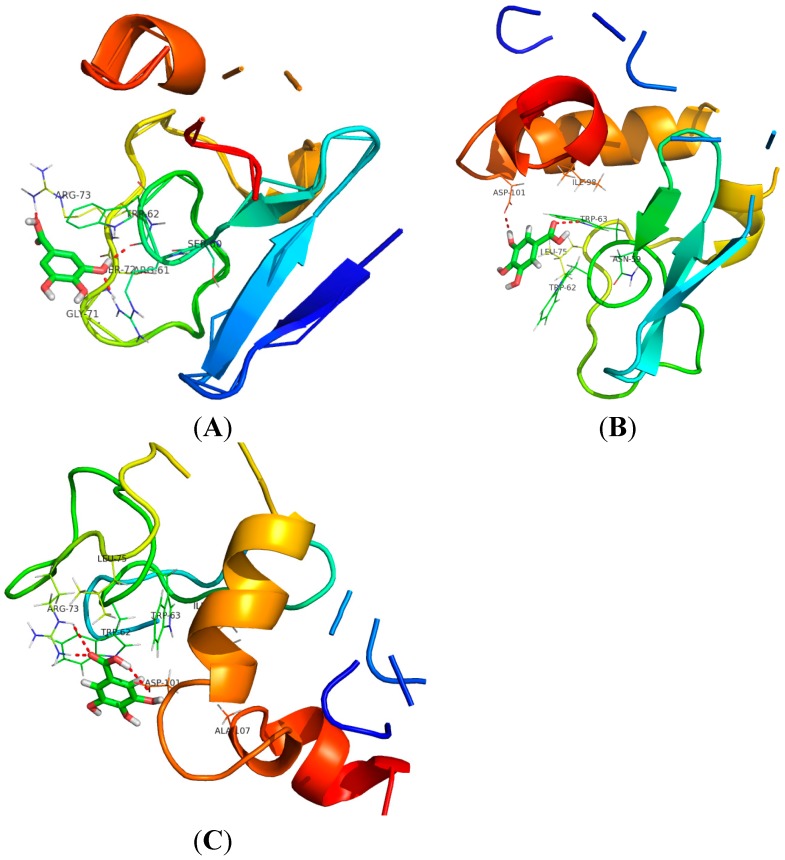
Interaction of GA with LYS, only residues around 21.2 Å of the ligand is displayed. (**A**) The interaction mode between GA and LYS before MD simulation; (**B**) The interaction mode between GA and LYS after 298 K MD simulation; (**C**) The interaction mode between GA and LYS after 310 K MD simulations. The ligand structure is represented using a green stick model, where the red sticks within represent oxygen. The hydrogen bond between the ligand and the protein is represented using a red dashed line.

The spectroscopy results showed that Trp residues in the binding pocket of LYS had obvious interactions with GA. The average structure of the GA-LYS complex during the 2 ns MD simulation was determined from the trajectory file. The binding models of GA-LYS were constructed by Pymol software and Discovery studio, and are shown in Figure 9, Figure 10 and Figure 11. Figure 9 shows the distance map of GA to Trp62 of LYS before and after MD simulation. Figure 10A shows the electron density and hydrophobic surface map between GA and LYS for the residues within 20.0 Å of the ligand. Figure 10B and Figure 11A show models of the initial GA-LYS complex before MD simulation. Figure 11B,C show the interaction mode between GA and LYS after 298 and 310 K MD simulations, respectively.

Figure 9 illustrates that the distance between GA and Trp62 get smaller after MD simulation and the binding distance between GA and Trp62 gets longer as the temperature gets warmer, which explains why the binding constant may decrease with increasing temperature.

The spectroscopy results show that the microdomain of Trp residues obviously interacts with GA, and GA binding to LYS can be seen from the molecular electron density map surrounding the ligand GA. GA molecules embed in the active surface pocket of LYS (Figure 10), which is mainly composed of the hydrophobic residues Ala107, Pro70, Pro79, Leu75, Trp62, Trp63, Ile58, Ile78, and Ile98. The spectroscopy results showed that hydrogen bonds and van der Waals force are the main driving forces to stabilize the GA-LYS interaction. The docking results confirmed that GA binds to LYS mainly by hydrogen bonding and hydrophobic interactions. The GA molecule is very close to the LYS Trp residues (Trp62 and Trp63) and Tyr residues (Tyr53), as shown in Figure 10B, which explains why GA can quench the endogenous fluorescence of LYS.

The distance between LYS (the acceptor) and GA (the donor) was 2.12 nm from the fluorescence quenching experiment. So we show the residues within 21.2 Å of GA in Figure 11A, and do not change the kernel structure and interaction force of GA. There is a large hydrophobic cavity in LYS to accommodate the drug molecule, and it plays an important role in the absorption, metabolism, and transportation of LYS. The GA benzene ring is embedded in the bonded zone, and the LYS amino acids Trp62, Trp63, and Tyr53 indicate the stability of the protein-drug system. Furthermore, there are a number of specific hydrogen bonds because several ionic and polar residues in the proximity of the ligand play an important role in stabilizing the complex via hydrogen bonds. Figure 11 shows the molecular docking domain before and after GA binds to LYS. Figure 11A shows the interaction mode between GA and LYS before the MD simulation. There are hydrogen-bond interactions between the hydroxyl oxygen atom of the carboxylic acid group of GA and Arg73, the 5-hydroxyl group and Trp62, and the oxygen atom of the 5-hydroxyl group and Arg61. Figure 11B shows the interaction between GA and LYS at the end of the 298 K MD simulation. There are hydrogen bonds between the carbonyl oxygen atom of the carboxylic acid of GA and Trp63, and the hydrogen atom of the 5-hydroxyl group and Asp101. Figure 11C shows the interaction between GA and LYS at the end of the 310 K MD simulation. There are hydrogen bonds between the carbonyl oxygen atom of the carboxylic acid group of GA and Arg73, and hydroxyl hydrogen atom of the carboxylic acid group of GA and Asp101. From the 2 ns MD trajectories analysis, we were able to detect the main H bonds between GA and LYS (Table 3). The docking conformations of GA-LYS were compared before and after the MD simulation. The amino acid residues around the active docking domain changed during the MD simulation, and the amino acid residues of LYS binding with GA were different before and after the MD simulation. The formation of hydrogen bonds formed between GA and Arg63, Arg73, Asp101 stabilizes the GA-LYS system. Therefore, it can be concluded that the interaction between GA and LYS is dominated by hydrophobic forces and hydrogen bonds, which agrees well with the binding mode observed by the fluorescence quenching mechanism of LYS in the presence of GA.

**Table 3:** H bonds formed between GA and LYS.

Temperatures (T/K)	Donor	Acceptor	Duration (% of the Total Simulation Time Considered)	Mean Distance (nm)	Mean Angle Degrees (°)
298	O1(GA)	O(Trp63)	7.20	2.808	18.63
	H5(GA)	O(Asp101)	3.40	2.974	33.08
310	O5(GA)	NH2(Arg73)	7.25	2.946	32.11
	H6(GA)	OD1(Asp101)	0.77	2.924	29.85

Actually, we have done the re-Dock verification to prove the LYS-GA molecular docking system believable. We extracted the ligand GA from the docked LYS-GA model and docked the after-extracted system with the original ligand 1,2-ethanediol (extracted from the LYS complex which was downloaded from the PDB database (coding 1GWD)). Then we compared the new compound with the original one (from the database). Analysis results showed that the binding domain of the 1,2-ethanediol with LYS stayed almost the same before and after the docking, so we believe that the docking methods in our article can be trusted.

### 2.7. MM-PBSA Free Energy

The relative binding free energies were calculated for the GA-LYS system with the MM-PBSA method. Table 4 shows the relative energy terms for the GA-LYS system at 298 and 310 K. The van der Waals energy and electrostatic interaction were the main contributors to the binding of GA to LYS. The total energies of the two systems were less than zero, which indicates that the complexes are stable at 298 and 310 K.

**Table 4:** Energy terms of MM-PBSA results for GA-LYS system at two different temperatures (*k*J/mol).

*T* (K)	Δ*G*_vdw_	Δ*G*_elec_	Δ*G*_pol-solv_	Δ*G*_n__onpol-solv_	Δ*G*_solv_	Δ*G*_gas_	Δ*G*
298	−44.92	−24.22	58.98	−1.79	57.20	−69.13	−11.93
310	−35.36	−52.48	81.42	−1.14	80.28	−87.84	−7.56

According to calculated results, the solvation free energy contributes little to the binding energy. The van der Waals (Δ*G*_vdw_) and electrostatic interactions (Δ*G*_elec_) are the main driving forces in the process of GA binding to LYS. The total Gibbs free energy change (Δ*G*) was −7.5551 *k*J/mol at 310 K, which is 4.3772 *k*J/mol higher than that at 298 K (−11.9323 *k*J/mol), showing that the binding tendency of GA to LYS decreases with increasing temperature. This indicates that temperature affects the binding of GA to lysozyme: the higher the temperature, the weaker the binding ability. The Δ*G* values calculated from the MM-PBSA method are negative and increase with increasing temperature, which is consistent with the spectroscopy results. However, because of the different calculation methods, the Δ*G* values are different. We believe that the calculated results under different MD conditions (target, pH, temperature, other parameters) are different, and our MM-PBSA calculated results are not very large under our conditions, but it is the theoretical calculated energies of the ligand-protein complex.

## 3. Experimental Section

### 3.1. Materials and Apparatus

Lysozyme (LYS, ≥18,000 U) was purchased from Sigma-Aldrich Company (Shanghai Co., Ltd., Shanghai, China). Gallic acid (GA, ≥97%) was purchased from Energy Chemical (Shanghai, China). The working solution of LYS (1.0 × 10^−5^ mol/L) and GA (1.0 × 10^−3^ mol/L) was prepared by dissolving them in Tris-HCl buffer solution of pH 7.43. Double distilled water was used in all solutions and the other chemicals used were analytical reagent.

All fluorescence spectra were recorded with a F-7000 spectrofluorophotometer (Hitachi, Co., Ltd., Tokyo, Japan) equipped with 1.0 cm quartz cells and a SLZC-10 thermostat bath (Shunliu Instrument Corporation, Nanjing, China). An UV-2450 UV-vis spectrometer (Shimadzu, Co., Kyoto, Japan) was used to record the scanning UV-visible spectra. The sample masses were accurately weighed using a BS224S electronic balance (Sartorius, Beijing, China).

### 3.2. Absorption and Fluorescence Spectroscopy

The absorption spectra of the LYS with concentrations of GA from 0.0 to 5.6 × 10^−5^ mol/L were recorded at room temperature. The wavelength range was 200–500 nm. The fluorescence measurements were performed at 291 and 310 K. The emission spectra were recorded in the wavelength range 250–500 nm upon excitation at 282 nm with a scanning speed of 240 nm/min. The widths of both the excitation slit and the emission slit were set to 2.5 nm. The spectrum of a blank sample containing only the buffer was used to correct for the fluorescence background.

### 3.3. Synchronous Fluorescence Spectroscopy and Fluorescence Phase Diagram

The synchronous fluorescence spectra were measured by simultaneously scanning the excitation and emission with a fixed wavelength difference (Δλ) between excitation and emission wavelengths. When Δλ was 15 or 60 nm, the synchronous fluorescence spectrum gave the characteristic information about tyrosine (Tyr) or tryptophan (Trp) residues. The fluorescence intensity data was extracted to draw the fluorescence phase diagram of GA binding to LYS.

### 3.4. Molecular Docking and Molecular Dynamics Simulations

The three-dimensional structure of LYS in complex with its 1,2-ethanediol ligand was obtained from the Protein Data Bank (PDB ID: 1GWD) (http://www.pdb.org). All the C1, I, Na and CO atoms were removed by Autodock program. After correcting atom types and adding all the hydrogen atoms, molecular building was done for GA ligand with molecular sketch program based on the structure of 1,2-ethanediol ligand. The ESP CHELPG charges calculated from B3LYP/6-31G* by Gaussian 98 was used for GA. RESP charges parameters were used for all LYS residues. Atomic partial charge generation and assignment of the force field were performed using Antechamber suite. Docking studies were performed with the AutoDock 4.02 suite of programs, and all of the calculations were performed on a Silicon Graphics Octane 2 workstation. 

Molecular dynamics simulations were carried out on the GA-LYS system using the SANDER module of AMBER11 with the Amber FF03 and GAFF force fields. The atomic charges and force field parameters of GA were added into the Amber FF03 force field to generate the topology file and coordinate file. The initial structure was placed in a truncated octahedral periodic box of TIP3P water molecules. We used the periodic boundary conditions and the distance between water box edges and the closest atom of the solutes was at least 10 angstrom. All of the simulations were carried out at neutral pH, and an adequate number of Cl ions were added to a simulation box in order to preserve neutrality. The system was minimized with the SANDER module with constant volume by 500 cycles of steepest descent minimization followed by 500 cycles of conjugate gradient minimization. These procedures ensured that the initial experimental structure was maintained while the solvent was allowed to relax. The steps above all featured 2000 cycles of steepest descent followed by conjugate gradient minimization. After energy minimization, canonical ensemble (NVT)-MD was carried out for 100 ps, during which the system was heated from 0 to 298/310 K. A 10 Å cut-off was set for the GA-LYS system. Finally, a 2 ns isothermal isobaric ensemble (NPT)-MD simulation was performed without any constraints. The time-step for the MD simulation was 2 fs, and every 2 ps a track file was recorded. The MD simulations of GA binding to LYS were analyzed using the Discovery Studio software package, followed by Origin8.0 and Pymol software.

### 3.5. MM-PBSA Method

The MM–PBSA method was used to calculate the binding free energy: ∆*G*_bind_ = *G*_complex_ − (*G*_receptor_ + *G*_ligand_), where *G*_complex_, *G*_receptor_, and *G*_ligand_ are the free energies of the complex, protein, and ligand, respectively. Each of the Δ*G* terms was calculated by summing the molecular mechanics free energy (Δ*G*_MM_), solvation free energy (Δ*G*_sol_), and vibrational entropy terms (*T*Δ*S*) [58]: Δ*G* = Δ*G*_MM_ + Δ*G*_sol_ − *T*Δ*S*. Δ*G*_MM_ is the standard force field energy, including strain energies from covalent bonds and torsion angles as well as noncovalent van der Waals and electrostatic energies. The solvation free energy (Δ*G*_sol_) was calculated with a PBSA model, which divides the solvation free energy into an electrostatic component (Δ*G*_pol, sol_) and a nonpolar component (Δ*G*_nonpol, sol_). We chose a total number of 50 snapshots from the last 500 ps on the trajectory with a regular interval of 10 ps. In this work, the binding free energy was calculated using MM-PBSA supplied with the AMBER11 package.

## 4. Conclusions

In this paper, we used fluorescence and UV-vis absorption spectroscopy together with MD simulations to investigate the interaction between GA and LYS. The fluorescence spectroscopy results showed that the quenching of LYS by GA is a result of the formation of the LYS-GA complex. GA can strongly bind to LYS, and the binding strength decreased with increasing temperature. Thermodynamic analysis showed that the binding forces between GA and LYS are mainly hydrogen bonding and van der Waals interactions. A synchronous fluorescence spectroscopy and fluorescence phase diagram method showed that the interaction of GA with LYS affects the conformation of the Trp residue microregion and the conformation change of LYS follows a two-state mode.

Molecular dynamics simulations were performed on the GA-LYS complex to investigate the binding mode of the complex. The mechanism of GA binding to LYS was revealed at the molecular level. The RMSD and RMSF values showed that the temperature affects the binding of GA to LYS: the lower the temperature, the smaller the protein flexibility. Molecule docking studies confirmed the interaction and microbinding domain between LYS and GA, and showed that the interaction was dominated by hydrogen bonding, van der Waals forces, and hydrophobic interactions, which is consistent with the spectroscopy experiments. From the MM–PBSA method, the complex was stable after MD simulation and the binding of GA to LYS was mainly by van der Waals forces and electrostatic interactions. We compared the free energy data obtained by the two different thermodynamic parameter calculation methods, and discovered that the free energy increased with increasing temperature, indicating that that the binding ability of GA to LYS at 310 K was weaker than at 298 K.

Due to the limited conditions of MD calculation in our experiment, the MD simulation method was firstly used in GA binding with LYS field, and our results showed that the hydrogen bonds between ligand and enzyme do not look very stable, perhaps because our primary MD results were not long enough for further calculation. Though the MD time may not be long enough, it can provide a primary reference for the researchers in the same interactional field. A long-playing MD simulation should obtain a stable state between ligand and acceptor.

Spectroscopy and molecular dynamics simulation all revealed that GA can interact with LYS to form a stable complex. Experimental error coupled with the theoretical calculation is considered under an ideal state lead to the experimental and theoretical results different. The experimental data was introduced into the Van’t Hoff equation to obtain Δ*G*°. The theoretical gists of the two calculative methods are different, so it is normal that the final results are different, but the results trend is roughly consistent. The molecular simulation results were almost consistent with spectroscopy. These results provide important insight into the interaction and mechanism of GA binding to LYS, which may be a useful guide for further pharmacological investigations.
